# Prebiotic effects of diet supplemented with the cultivated red seaweed *Chondrus crispus* or with fructo-oligo-saccharide on host immunity, colonic microbiota and gut microbial metabolites

**DOI:** 10.1186/s12906-015-0802-5

**Published:** 2015-08-14

**Authors:** Jinghua Liu, Saveetha Kandasamy, Junzeng Zhang, Christopher W. Kirby, Tobias Karakach, Jeff Hafting, Alan T. Critchley, Franklin Evans, Balakrishnan Prithiviraj

**Affiliations:** Department of Environmental Sciences, Dalhousie University, Truro, NS B2N 2R8 Canada; Natural Products Chemistry, National Research Council of Canada, Halifax, B3H 3Z1 Canada; Crops and Livestock Research Centre, Agriculture and Agri-Food Canada, Charlottetown, PE C1A 4N6 Canada; Acadian Seaplants Limited, 30 Brown Ave., Dartmouth, NS B3B 1X8 Canada

## Abstract

**Background:**

Gastrointestinal microbial communities are diverse and are composed of both beneficial and pathogenic groups. Prebiotics, such as digestion-resistant fibers, influence the composition of gut microbiota, and can contribute to the improvement of host health. The red seaweed *Chondrus crispus* is rich in dietary fiber and oligosaccharides, however its prebiotic potential has not been studied to date.

**Methods:**

Prebiotic effects were investigated with weaning rats fed a cultivated *C. crispus*-supplemented diet. Comparison standards included a fructo-oligo-saccharide (FOS) diet and a basal diet. The colonic microbiome was profiled with a 16S rRNA sequencing-based Phylochip array. Concentrations of short chain fatty acids (SCFAs) in the feacal samples were determined by gas chromatography with a flame ionization detector (GC-FID) analysis. Immunoglobulin levels in the blood plasma were analyzed with an enzyme-linked immunosorbent assay (ELISA). Histo-morphological parameters of the proximal colon tissue were characterized by hematoxylin and eosin (H&E) staining.

**Results:**

Phylochip array analysis indicated differing microbiome composition among the diet-supplemented and the control groups, with the *C. crispus* group (2.5 % supplementation) showing larger separation from the control than other treatment groups. In the 2.5 % *C. crispus* group, the population of beneficial bacteria such as *Bifidobacterium breve* increased (4.9-fold, p = 0.001), and the abundance of pathogenic species such as *Clostridium septicum* and *Streptococcus pneumonia* decreased. Higher concentrations of short chain fatty acids (i.e., gut microbial metabolites), including acetic, propionic and butyric acids, were found in faecal samples of the *C. crispus*-fed rats. Furthermore, both *C. crispus* and FOS supplemented rats showed significant improvements in proximal colon histo-morphology . Higher faecal moisture was noted in the 2.5 % *C. crispus* group, and elevated plasma immunoglobulin (IgA and IgG) levels were observed in the 0.5 % *C. crispus* group, as compared to the basal feed group.

**Conclusions:**

The results suggest multiple prebiotic effects, such as influencing the composition of gut microbial communities, improvement of gut health and immune modulation in rats supplemented with cultivated *C. crispus*.

**Electronic supplementary material:**

The online version of this article (doi:10.1186/s12906-015-0802-5) contains supplementary material, which is available to authorized users.

## Background

Mammalian gastrointestinal (GI) tracts are colonized by a large number of beneficial and pathogenic microbes. There are up to 10^12^ bacteria per gram of digesta in the colon, the most intensively colonized portion of the GI tract [1]. Beneficial gut bacteria (i.e., probiotics), such as *Bifidobacterium spp.* and *Lactobacillus spp.* promote animal and human health by providing nutritional metabolites, such as short chain fatty acids, through selective fermentation of digestion-resistant carbohydrates [2]. The digestion-resistant carbohydrates (i.e., prebiotics), serve as a nutrient source for probiotic bacteria. Dietary supplementation of prebiotics and/or probiotics can help to maintain a favorable ratio of the beneficial to harmful bacteria in the host gut, allowing competition to favor beneficial groups [2, 3].

Research shows that consumption of prebiotics and/or probiotics results in multiple health benefits including improving consistency and frequency of the stool [4, 5], preventing allergic diseases [6–8], regulating immunity [9–11] and decreasing the risks of cancers [12, 13] and obesity [8, 12]. For example, when children with food allergy-related severe atopic eczema were fed *Lactobacillus rhamnosus* and *Bifidobacterium lactis*, they showed significant improvement in clinical symptoms, as compared to the placebo group [14, 15]. Likewise, in formula-fed infants, IgA immune response was reported to be enhanced both by *B. lactis* and *L. rhamnosus* [16]. In addition, consumption of milk fortified with prebiotic oligosaccharide and the probiotic bacterium *B. lactis* was reported to reduce episodes of dysentery, pneumonia, and severe acute lower respiratory infection (as compared to the placebo group), in 1–4 year old children, who had limited access to hygienic conditions [17].

Although the mechanisms underlying the health promoting benefits of pre/probiotics are not well understood, recent *in vitro* research suggests that effects on the function of intestinal epithelial cells and mucosal immunity are important. For example, a probiotic combination of *Bifidobacterium spp.*, *Lactobacillus spp.*, and *Streptococcus salivarius* decreased the permeability of the intestinal epithelial cells and thus increased the barrier function [18]. Additionally, *S. thermophilus* and *L. acidophilus* were shown to prevent adhesion and invasion by pathogenic entero-invasive *Escherichia coli* and epithelial dysfunction, accompanied by maintenance and/or enhancement of cytoskeletal and tight junctional protein phosphorylation [19]. Moreover, epithelial cell-mediated immunity was induced by probiotic bacteria such as Lactobacilli, and this immune modulatory effect was shown to be strain specific [20].

The red seaweed *Chondrus crispus* (Rhodophyta) is widely distributed in the northern Atlantic. As an economically important seaweed species in the Atlantic Canada region, *C. crispus* is also cultivated on land in Nova Scotia, Canada. In addition to high content of total proteins, oligopeptides and pigments, this alga is rich in the water-soluble polysaccharide carrageenan (approximately 50-65 % on a basis of dry weight) [21, 22]. Carrageenan is widely used in the food industry as a thickener, stabilizer and emulsifier. This high polysaccharide content suggests that *C. crispus* may be a rich source of prebiotic fiber. However, *C. crispus* has not yet been investigated for its prebiotic potential. In the current study,the prebiotic effects of cultivated *C. crispus* supplemented in the feed of rats wasinvestigated. Prebiotic parameters such as composition and activity of colonic microbiota, host immunity and selective metabolites, colon histological morphology and faecal moisture were measured.

## Methods

### Preparation of diets

Mechanically air-dried, whole plants of a proprietary strain of *Chondrus crispus*, cultivated intensively on-land, were obtained from Acadian Seaplants Limited (Dartmouth, Nova Scotia, Canada). The seaweed samples were finely ground and passed through a 60-mesh sieve (0.25 mm in diameter). The fructo-oligo-saccharide (FOS) powder, extracted from chicory roots, was provided by Cargill (Wayzata, MN), as Oliggo – Fiber™ DS2 inulin, with an average degree of polymerization less than 10. *C. crispus* and FOS were mixed with a standard basal feed (RMH 3000, LabDiet, St. Louis, MO, USA), respectively, at the ratio of 0.0 (plus 2.5 % corn starch), 0.5 (plus 2.0 % corn starch) or 2.5 % (dry w/w). The mixed feed was then pelleted (4.7 mm in diameter, 1.0-1.5 cm in length) using a feed mill facility located at the Faculty of Agriculture, Dalhousie University, Truro, Nova Scotia. Diets were prepared just before the trial and stored under dry and cool conditions.

### Animals and sampling procedures

All animal protocols were approved by the University Committee on Laboratory Animals at Dalhousie University, Canada. Male Sprague–Dawley rats (21 days old; Charles River Laboratories Inc., Montreal, Canada) were individually housed in standard plastic cages with a 12-hour light–dark cycle, at 22 +/− 2 °C, with free access to food and water. Rats were randomly assigned to each of the five feeding groups (n = 6/group). Feed groups consisted ofthe basal diet control group (BF), the basal diet supplemented with 2.5 or 0.5 % (dry w/w) of cultivated *Chondrus* (C2.5 and C0.5, respectively), and the basal diet supplemented with 2.5 or 0.5 % (dry w/w) of FOS (F2.5 and F0.5, respectively). Environmental enrichment was provided by 50 mm diameter wooden pieces and 100 mm diameter plastic tubes. Feed intake and body weight for each animal were monitored weekly. After 21 days of feeding, fresh faeces were collected. One fecal portion was weighed immediately, dried thoroughly at 70 °C (for 24 h) and analyzed for moisture content. Another fecal portion was immediately frozen in liquid nitrogen and stored at −80 °C until further analysis. Rats were anesthetized with 3 % isoflurane, and euthanized by blood draining through cardiac puncture. Blood samples were collected into anticoagulant (K_2_-EDTA)-coated 5-ml tubes (BD, USA), and immediately centrifuged at 5000 × g for 15 min. The resulting plasma was stored at −20 °C until analysis. Liver, kidney, spleen and heart were collected, blotted on filter paper and weighed. Colon contents from each animal were squeezed into sterile micro-centrifuge tubes, snap-frozen and stored at −80 °C. The colon tissue was flushed with 0.9 % NaCl, and a 0.5-cm segment, excised 1-cm from the end of the proximal colon, was soaked in 10 % neutral, buffered formalin solution (Sigma).

### Immunoglobulin enzyme-linked immunosorbent assay (ELISA)

The frozen plasma samples were thawed on ice and subjected to IgA and IgG assays as previously described [23], using the rat IgA and IgG ELISA kits, respectively (Genway, San Diego, USA), following the manufacturer’s instructions. Three technical replicates were performed for each plasma sample collected. Optical density was read on a microplate reader (BioTek) at 450 nm. A standard curve generated from serial dilution of the rat IgA or IgG, of a known concentration, as provided in the kit, was used to determine the sample concentration of IgA and IgG, respectively.

### Colonic Histomorphology

After being fixed for 3 days in 10 % neutral formalin, the proximal colon samples were paraffin embedded, sectioned transversely and subjected to hematoxylin and eosin (H&E) staining, following standard procedures [24]. For each H&E section, at least 30 bright-field images were captured by a Motic 2500 digital camera (Motic, China), under 40–100 × magnification, using an Olympus BHS microscope (Olympus, Japan). The measurement function of the Motic Images Plus 2.0 ML software (Motic, China) was used to determine colonic crypt depth, and the thickness of the colonic mucosa, externa muscularis and total wall.

### Bacterial DNA isolation from colon content

Bacterial DNA was extracted from 200 mg of colon digesta using the QIAamp DNA Stool Mini Kit (Cat # 51504, Qiagen, USA) following the supplier’s instructions. The DNA was quantified with a Nanodrop ND-2000 spectrophotometer (NanoDrop Technologies Wilmington, DE), and the integrity of DNA was determined by agarose gel electrophoresis. The DNA was stored at −20 °C until further analysis.

### Colonic microbiota profiling and analyses

Colonic microbiota analysis was carried out using PhyloChip Arrays (Second Genome Inc., CA, USA). No less than 200 ng of colonic bacterial DNA (n = 4/group) was used as the template for bacterial 16S rRNA gene amplification. The PCR was run for 35 cycles at 95 °C for 30 sec for denaturing, 50 °C for 30 sec for annealing, and 72 °C for 2 min for extension, using the Ex Taq system (Takara Bio Inc., Japan). Primer sequences are as follows: forward primer, 5´-AGRG TTTG ATCM TGGC TCAG-3´; reverse primer, 5´-GGTT ACCT TGTT ACGA CTT-3´. The resulting PCR product from each sample was concentrated and quantified by electrophoresis using an Agilent 2100 Bioanalyzer (Agilent Technologies, CA, USA). PhyloChip Control Mix (Second Genome Inc.) was then added to label the PCR products, which were then fragmented, biotin labeled and hybridized to the G3 PhyloChip Array. PhyloChip arrays were washed, stained and scanned using a GeneArray scanner (Affymetrix, OH, USA) and each scan was captured using the GeneChip Microarray Analysis Suite (Affymetrix). The hybridization score derived from the fluorescence intensity (FI) (the mean log_2_ FI × 1,000) for each operational taxonomic unit (OTU) was used to denote abundance. OTUs were defined by >99 % similarity of the 16S rRNA sequence. A threshold based on perfect match and mismatch intensities of multiple probes per probe set [25] was used to determine the presence/absence of an OTU. Taxa-sample intersections were analyzed based on the abundance (AT) and binary matrices (BT). The Unifrac distance metric [26] and weighted Unifrac distance metric were used to compute the pairwise BT and AT dissimilarity scores; the weighted Unifrac metric reflects the abundance of and the phylogenetic distance between OTUs. Hierarchical clustering via average-neighbor (HC-AN) and principal coordinate analysis (PCA) were used to graphically summarize inter-sample relationships on the basis of AT and BT dissimilarity scores. Thereafter, we analyzed the abundance data and identified the taxa which showed significant/greatest changes between the control and the treated groups.

### Gas chromatography analysis of short chain fatty acids (SCFAs)

The gut microbial metabolites, SCFAs, in rat faecal samples were quantified by gas chromatography (GC) following a previously described protocol [27], with modifications. A Bruker 430-GC system (Billerica, MA, USA), equipped with a flame ionization detector (FID) and an automatic liquid sampler, was used. A J&W DB-FFAP capillary column (Agilent Technologies Inc., USA; Part # J125-3232, 30 m × 0.53 mm × 1-μm film thickness) was used. An aliquot of 0.2 g of faeces, which was previously frozen and thawed on ice, was homogenized in 2 ml of extraction buffer (0.1 % (w/v) HgCl_2_ and 1 % (v/v) H_3_PO_4_) containing 0.045 g/l of 2-ethylbutyric acid (Sigma) as an internal standard. The resulting slurry was centrifuged, prior to the supernatant being passed through a 0.2 μm filter. The injection volume was 0.5 μl. Each sample run was preceded with a wash run of 1 % formic acid. The oven temperature was held at 80 °C for 1.2 min, then increased to 200 °C at 10 °C /min and held for 5 min. The temperature for the FID and the injection port was 220 °C and 180 °C, respectively. The flow rate of helium, hydrogen, and air was 25, 30, and 300 ml/min, respectively. Specific SCFAs were identified by running an external volatile acid standard mix (Supelco, USA). The concentration of SCFAs was quantified by running the internal standard and the external standard mix, as previously described [28]. All reagents were of GC grade and solutions were prepared with deionized water.

### Statistics

The statistical analyses were performed using SPSS 15.0 (SPSS, USA). Data were presented as the mean ± SD or SE. Differences between the control group and the dietary supplemented groups were assessed using one-way ANOVA followed by the independent two-tailed *t* test or the Mann–Whitney test. Differences were considered as significant when p < 0.05.

## Results

### Effect of diets on body weight, organ weight, faecal moisture, and host immunity

**Fig. 1:**
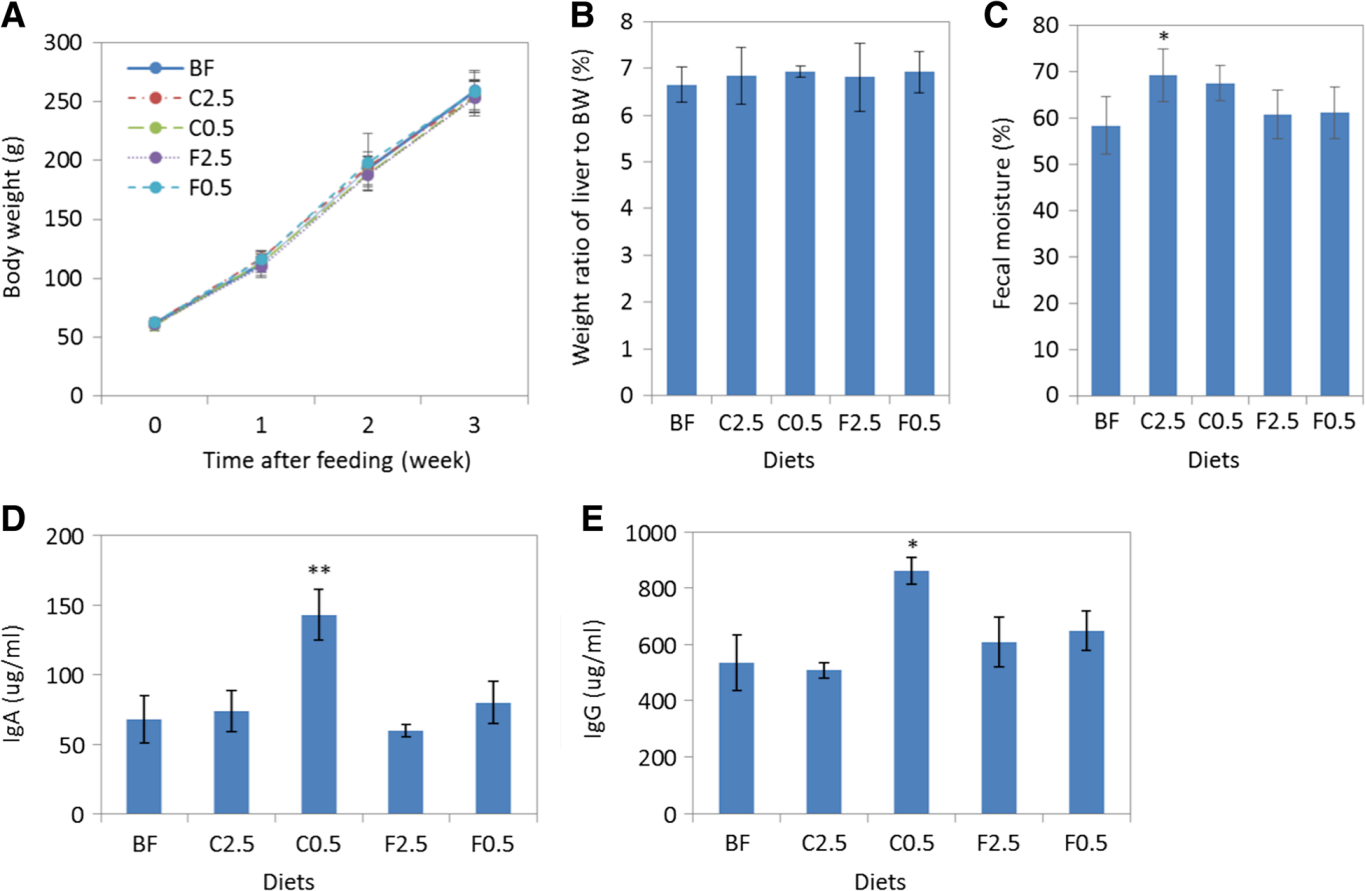
Effect of diets on body weight gain, liver weight, faecal moisture, and plasma immunoglobulin. Body weight (**a**) was monitored weekly during the feeding period. Liver weight (**b**) and faecal moisture (**c**) were determined after 3 weeks of feeding. Data are presented as the mean ± SD (n = 6). At the end of the feeding period, IgA (**d**) and IgG (**e**) levels in rat plasma samples were determined by ELISA and are presented as the mean ± SD, with three biological replicates and two technical replicates. BF = basal feed; C2.5 = *C. crispus* 2.5 %; C0.5 = *C. crispus* 0.5 %; F2.5 = FOS Inulin 2.5 %; F0.5 = FOS Inulin 0.5 %. *p < 0.05; **p < 0.01 (versus BF)

Dietary supplementation with either cultivated red seaweed *C. crispus* or FOS did not affect rat body weight gain (Fig. 1a). There was also no effect on the weight of organs such as kidney, heart and spleen (Additional file 1). Although there was a minor increase in the weight of liver in all fiber-supplemented groups, the change was not statistically significant (Fig. 1b) as compared to the control rats.The *C. crispus* supplemented diet C2.5 increased faecal moisture after 21 days of feeding (increased by 17 % (p = 0.04)), as compared to the control group (Fig. 1c). The C0.5, F0.5 andF2.5 diets did show a slight increase in faecal moisture, but it was not statistically significant.

Based on our previous observations on the immune-modulation effect of *C. crispus* [29], immunoglobulin levels in rats were expected to be influenced by dietary *C. crispus*. The IgA level in the C0.5 group (143 μg/ml) increased 2-fold, as compared to the control group (p = 0.006) (Fig. 1d). Likewise, the IgG level in the C0.5 group increased 1.6-fold over the the control group (p = 0.02) (Fig. 1e). Thus, both IgA and IgG levels were significantly elevated in the group fed with the lower rate of *C. crispus* (C0.5), as compared to the basal feed group (Fig. 1).

### Effect of diets on the overall composition of colonic microbiota

**Fig. 2:**
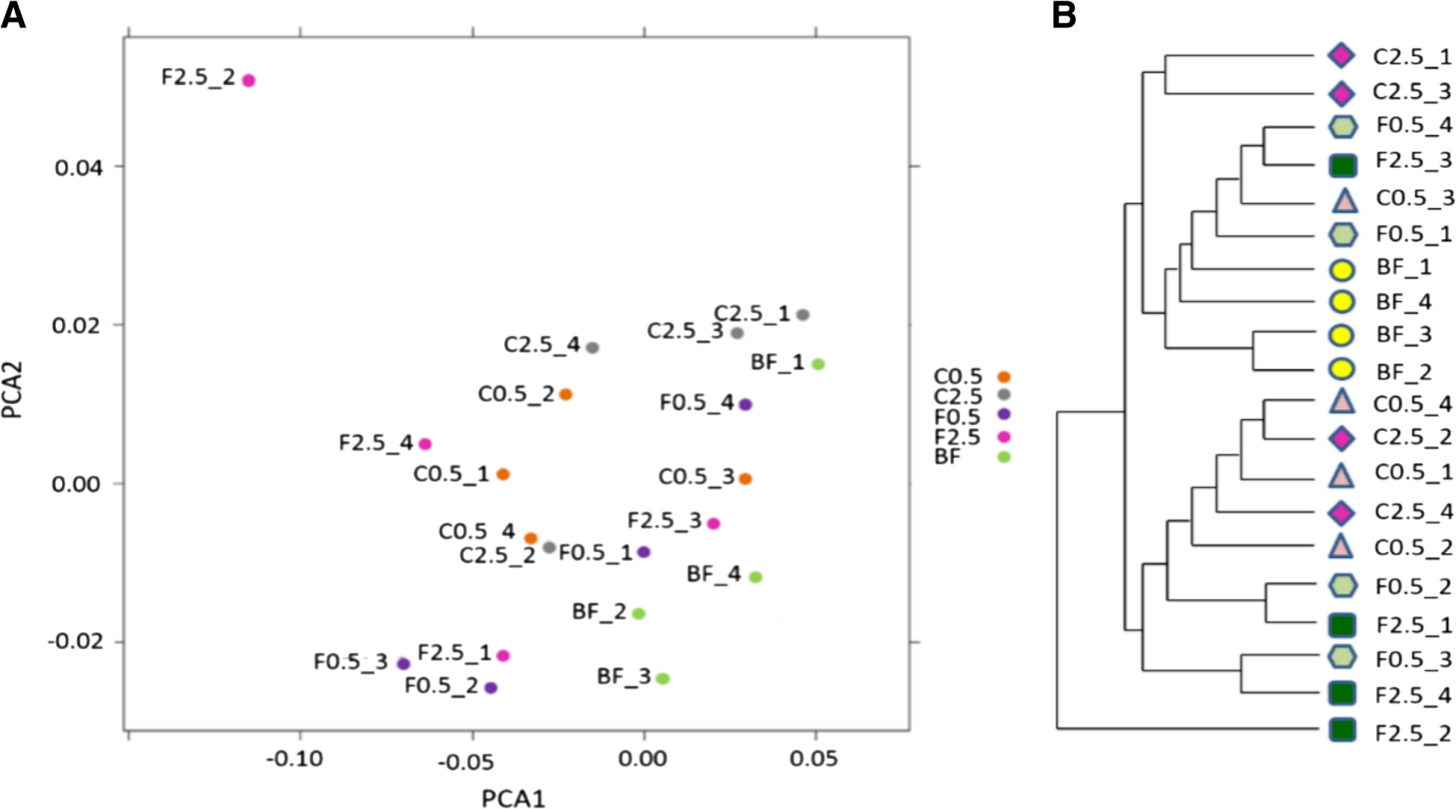
Effect of diets on the structure and composition of the colonic microbiome. **a** PCA analysis based on weighted Unifrac distance among the samples of 906 taxa with significant abundance differences across at least one of the groups. PCA1, 54 % of variation; PCA2, 11 % of variation. Larger variation from the basal feed group represents greater dissimilarity. **b** HC-AN analysis of hierachical clustering based on weighed Unifrac distance between samples given abundance of 906 taxa present in at least one of the samples. In the dentrogram, components clustering with greater distance shows greater dissimilarity. PCA, principal component analysis. BF = basal feed; C2.5 = *C. crispus* 2.5 %; C0.5 = *C. crispus* 0.5 %; F2.5 = FOS Inulin 2.5 %; F0.5 = FOS Inulin 0.5 %

Ingestion of non-digestible carbohydrates can differentially stimulate the growth of colonic bacterial groups [2]. Therefore, colonic microbiota profiling was used to investigate the prebiotic potential of non-starch, polysaccharide-rich, cultivated *C. crispus*. Through principal component analysis (PCA), a general differentiation between the supplemented diets and the control diet was evident (Fig. 2a). Larger variation from the basal feed group indicated greater dissimilarity. Additionally, the HC-AN analysis based on weighed Unifrac distance showed differential hierarchical clustering between the control group and the supplemented groups, especially for the C0.5 group (Fig. 2b).

### Effect of diets on the taxonomic composition of colonic microbiota

**Fig. 3:**
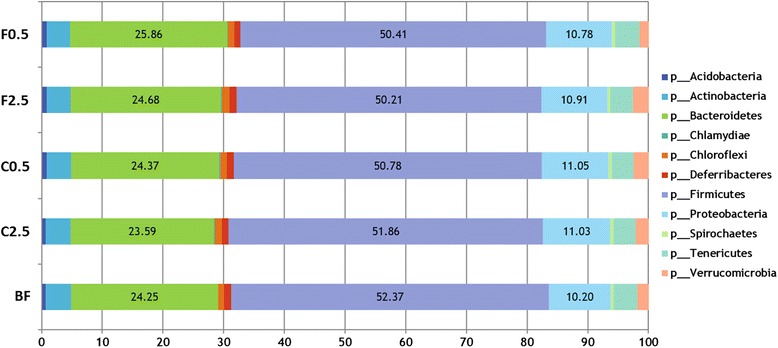
Effect of diets on colonic microbiota composition at the phylum level. Proportions of various phyla are shown as percentage of the total abundance of microbiota. BF = basal feed; C2.5 = *C. crispus* 2.5 %; C0.5 = *C. crispus* 0.5 %; F2.5 = FOS Inulin 2.5 %; F0.5 = FOS Inulin 0.5 %

**Fig. 4:**
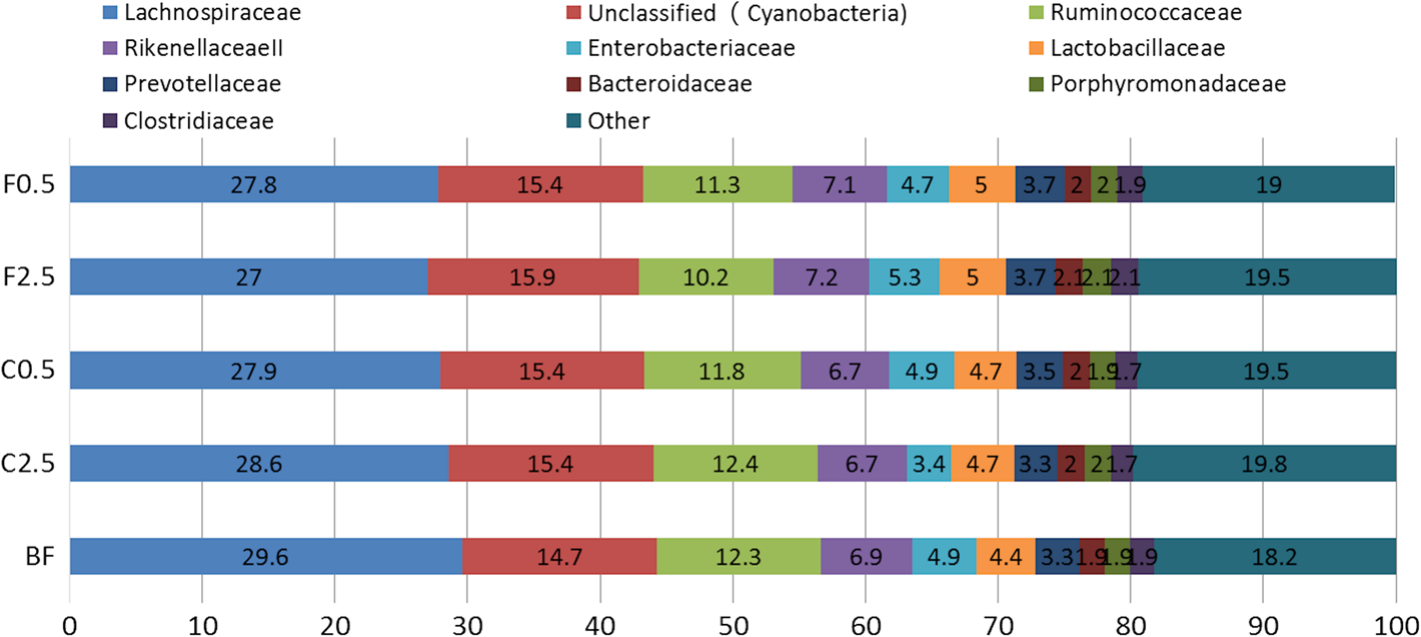
Effect of diets on colonic microbiota composition at the family level. Proportions of the top ten richest taxa at the family rank are shown as percentage of the total abundance of microbiota. BF = basal feed; C2.5 = *C. crispus* 2.5 %; C0.5 = *C. crispus* 0.5 %; F2.5 = FOS Inulin 2.5 %; F0.5 = FOS Inulin 0.5 %

Although colonic microbiota community composition was affected by diet (Fig. 2), its composition remains stable at the higher taxonomic levels of phylum and family, among the various diet groups. At the phylum level, the Firmicutes, Bacteroidetes and Proteobacteria predominated in the gut for all diet groups, accounting for 52.37 %, 24.25 %, and 10.2 %, respectively (Fig. 3). For each diet group, either control or supplemented, the ten most abundant families (Lachnospiraceae, Ruminococcaceae, RikenellaceaeII, Lactobacillaceae, Enterobacteriaceae, Prevotellaceae, Bacteroidaceae, Porphyromonadaceae, unclassified (Cyanobacteria) and Clostridiaceae) collectively account for an average of 81 % of the colonic microbiota. The microbiota family composition was not significantly affected by either diet (Fig. 4). At the genus level, the abundance of the identified genera was shifted differentially by the *C. crispus*- or FOS-supplemented diets (see Tables 1 and 2). With the C2.5 diet, there were abundance increases in 7 genera, including *Bifidobacterium* (p = 0.001), *Legionella* (p < 0.0001), *Sutterella* (p = 0.001), *Blautia* (p = 0.004), *Holdemania* (p = 0.032), *Shewanella* (p = 0.01) and *Agarivorans* (p = 0.005), together with a decrease in the abundance of the genus *Streptococcus* (p = 0.046), as compared to the control (Table 1). The C0.5 group showed no significant changes in these genera compared to the control. Dietary supplementation with 2.5 % FOS (F2.5) resulted in abundance shifts in a different set of genera (Table 2). For example, both F2.5 and F0.5 groups showed increased *Actinetobacter* (p = 0.011, 0.006, respectively) and *Pseudomonas* (p = 0.017, 0.012, respectively). In addition, the F2.5 diet was associated with increases in the community of *Alicyclobacillus* (p = 0.009) and *Bacillus* (p = 0.045), together with a decreased abundance of *Coprococcus* (p = 0.009) (Table 2).

**Table 1:** The 20 colonic bacterial genera with the greatest increase or decrease in abundance in rats fed a seaweed-supplemented diet (C2.5), and their abundance in other diet supplemented groups. BF = basal feed; C2.5 = *C. crispus* 2.5 %; C0.5 = *C. crispus* 0.5 %; F2.5 = FOS Inulin 2.5 %; F0.5 = FOS Inulin 0.5 %

Phylum	Class	Order	Family	Genus	Fold change^a^			
					C2.5	C0.5	F2.5	F0.5
Proteobacteria	Alphaproteobacteria	Rhizobiales	Aurantimonadaceae	Aurantimonas	11.7	12.5	4.7	7.8
Actinobacteria	Actinobacteria	Bifidobacteriales	Bifidobacteriaceae	Bifidobacterium	4.8	1.1	2	1.6
Proteobacteria	Gammaproteobacteria	Alteromonadales	Alteromonadaceae	Agarivorans	3.2	1.8	1.5	−1.8
Proteobacteria	Gammaproteobacteria	Legionellales	Legionellaceae	Legionella	2.9	2	2	1.3
Proteobacteria	Gammaproteobacteria	Alteromonadales	Shewanellaceae	Shewanella	2.6	1.5	−1.3	−1.9
Firmicutes	Clostridia	Clostridiales	Lachnospiraceae	Blautia	2.4	1.6	2.3	−2.5
Proteobacteria	Betaproteobacteria	Burkholderiales	Alcaligenaceae	Sutterella	2.0	1.6	2.4	1.1
Tenericutes	Erysipelotrichi	Erysipelotrichales	Erysipelotrichaceae	Holdemania	1.8	1.5	2.4	1.1
Firmicutes	Clostridia	Clostridiales	Lachnospiraceae	Dorea	1.6	1.1	−1.1	−1.2
Firmicutes	Bacilli	Bacillales	Staphylococcaceae	Staphylococcus	1.4	1.1	1.3	1.2
Actinobacteria	Actinobacteria	Actinomycetales	Micrococcaceae	Arthrobacter	−1.2	−1.1	−1.1	−1.2
Firmicutes	Clostridia	Clostridiales	Lachnospiraceae	Oribacterium	−1.3	1.0	−2.3	−2.0
Proteobacteria	Alphaproteobacteria	Rhodobacterales	Rhodobacteraceae	Thioclava	−1.4	−1.0	−2.6	−2.8
Actinobacteria	Actinobacteria	Actinomycetales	Micrococcaceae	Micrococcus	−1.6	−1.5	−1.3	−1.8
Firmicutes	Bacilli	Lactobacillales	Streptococcaceae	Streptococcus	−1.9	−1.3	−1.3	−1.4
Actinobacteria	Actinobacteria	Actinomycetales	Intrasporangiaceae	Janibacter	−2.0	1.2	−4.0	−1.2
Tenericutes	Mollicutes	Entomoplasmatales	Spiroplasmataceae	Spiroplasma	−2.1	−1.5	−1.5	1.2
Firmicutes	Clostridia	Clostridiales	Lachnospiraceae	Pseudobutyrivibrio	−2.3	−6.4	−1.3	1.1
Proteobacteria	Gammaproteobacteria	Enterobacteriales	Enterobacteriaceae	Proteus	−5.9	−1.3	1.6	1.0
Proteobacteria	Gammaproteobacteria	Enterobacteriales	Enterobacteriaceae	SMC	−24.4	−1.4	1.8	−1.0

^a^Fold change represents the relative abundance of the bacterial genus in rats fed seaweed- or inulin-supplemented diets compared with the basal diet; values were calculated from the microarray hybridization scores derived from fluorescence intensity. C2.5, *Chondrus crispus* 2.5 % (dry w/w); C0.5, 0.5 %; F2.5, FOS inulin 2.5 %; F0.5, 0.5 %

**Table 2:** The 20 colonic bacterial genera with greatest increase or decrease in abundance in rats fed a FOS inulin-supplemented diet (F2.5), and their abundance in other diet supplemented groups. BF = basal feed; C2.5 = *C. crispus* 2.5 %; C0.5 = *C. crispus* 0.5 %; F2.5 = FOS Inulin 2.5 %; F0.5 = FOS Inulin 0.5 %

Phylum	Class	Order	Family	Genus	Fold change^a^			
					C2.5	C0.5	F2.5	F0.5
Proteobacteria	Alphaproteobacteria	Rhizobiales	Aurantimonadaceae	Aurantimonas	11.7	12.5	4.7	7.8
Actinobacteria	Actinobacteria	Bifidobacteriales	Bifidobacteriaceae	Bifidobacterium	4.8	2.1	2.9	1.6
Firmicutes	Clostridia	Clostridiales	Lachnospiraceae	Blautia	2.4	1.5	2.3	−2.5
Proteobacteria	Gammaproteobacteria	Pseudomonadales	Moraxellaceae	Acinetobacter	−1.1	1.2	2.1	2.7
Proteobacteria	Gammaproteobacteria	Legionellales	Legionellaceae	Legionella	2.9	2.0	2.0	1.3
Tenericutes	Mollicutes	Entomoplasmatales	Entomoplasmataceae	Mesoplasma	1.3	1.4	1.9	1.8
Proteobacteria	Gammaproteobacteria	Enterobacteriales	Enterobacteriaceae	SMC	−24.4	−1.4	1.8	1.0
Proteobacteria	Gammaproteobacteria	Enterobacteriales	Enterobacteriaceae	Proteus	−5.9	−1.3	1.6	1.0
Chloroflexi	Anaerolineae	Anaerolineales	Anaerolinaceae	Anaerolinea	1.3	1.2	1.6	1.0
Chlamydiae	Chlamydiae	Chlamydiales	Parachlamydiaceae	Parachlamydia	1.2	1.5	1.6	−1.1
Firmicutes	Clostridia	Clostridiales	Ruminococcaceae	Oscillospira	1.2	1.0	−1.3	−1.3
Proteobacteria	Gammaproteobacteria	Alteromonadales	Shewanellaceae	Shewanella	2.6	1.5	−1.3	−1.9
Firmicutes	Clostridia	Clostridiales	Lachnospiraceae	Pseudobutyrivibrio	−2.3	−6.4	−1.3	1.1
Firmicutes	Bacilli	Lactobacillales	Streptococcaceae	Streptococcus	−1.9	−1.3	−1.3	−1.4
Firmicutes	Clostridia	Clostridiales	Ruminococcaceae	Ruminococcus	1.0	1.1	−1.3	1.0
Tenericutes	Mollicutes	Entomoplasmatales	Spiroplasmataceae	Spiroplasma	−2.1	−1.5	−1.5	1.2
Proteobacteria	Alphaproteobacteria	Rhizobiales	Rhizobiaceae	Rhizobium	1.0	1.1	−2.0	−1.9
Firmicutes	Clostridia	Clostridiales	Lachnospiraceae	Oribacterium	−1.3	1.0	−2.3	−2.0
Proteobacteria	Alphaproteobacteria	Rhodobacterales	Rhodobacteraceae	Thioclava	−1.4	1.0	−2.6	−2.8
Actinobacteria	Actinobacteria	Actinomycetales	Intrasporangiaceae	Janibacter	−2.0	1.1	−4.0	−1.2

^a^Fold change represents the relative abundance of the bacterial genus in rats fed seaweed- or inulin-supplemented diets compared with the basal diet; values were calculated from the microarray hybridization scores derived from fluorescence intensity. C2.5, *Chondrus crispus* 2.5 % (dry w/w); C0.5, 0.5 %; F2.5, FOS inulin 2.5 %; F0.5, 0.5 %

**Fig. 5:**
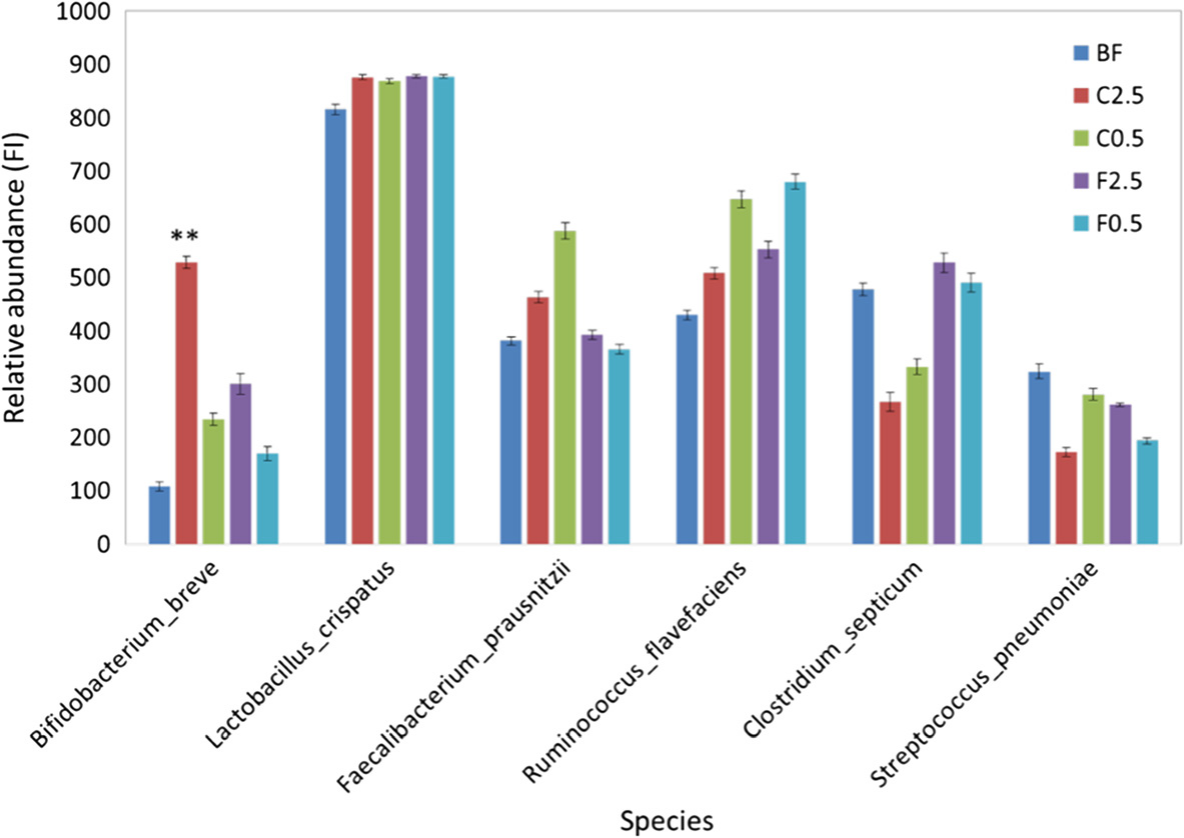
Effect of diets on the abundance of species of beneficial or pathogenic potential from the colonic microbiota. Relative abundance of the species was calculated by the fluorescence intensity (FI) derived from microarray hybridization scores. BF = basal feed; C2.5 = *C. crispus* 2.5 %; C0.5 = *C. crispus* 0.5 %; F2.5 = FOS Inulin 2.5 %; F0.5 = FOS Inulin 0.5 %. Data are presented as mean ± SE. **p < 0.01 (versus BF)

Among the identified species of colonic bacteria, diet affected the abundance of some beneficial, as well as pathogenic species (Fig. 5). Specifically, as compared to the control group, there was a 4.9-fold and 2.2-fold increase in the abundance of the well-established probiotic bacterium, *Bifidobacterium breve*, in the C2.5 (p = 0.001) and C0.5 (p = 0.15) groups. On the other hand, the F2.5 and F0.5 groups showed less enhanced abundance (p = 0.349, and 0.533, respectively) of *B. breve* (Fig. 5). Hence, the influence of *C. crispus* and FOS on the colonic *B. breve* community showed a dose-dependency, with the higher concentration associated with a greater abundance of *B. breve*. In contrast, the potentially-pathogenic species, *Clostridium septicum* and *Streptococcus pneumoniae* decreased slightly, in C2.5, C0.5, and F0.5 groups although these decreases with not statistically significant (Fig. 5).

### Effect of diets on the gut microbial metabolites

**Fig. 6:**
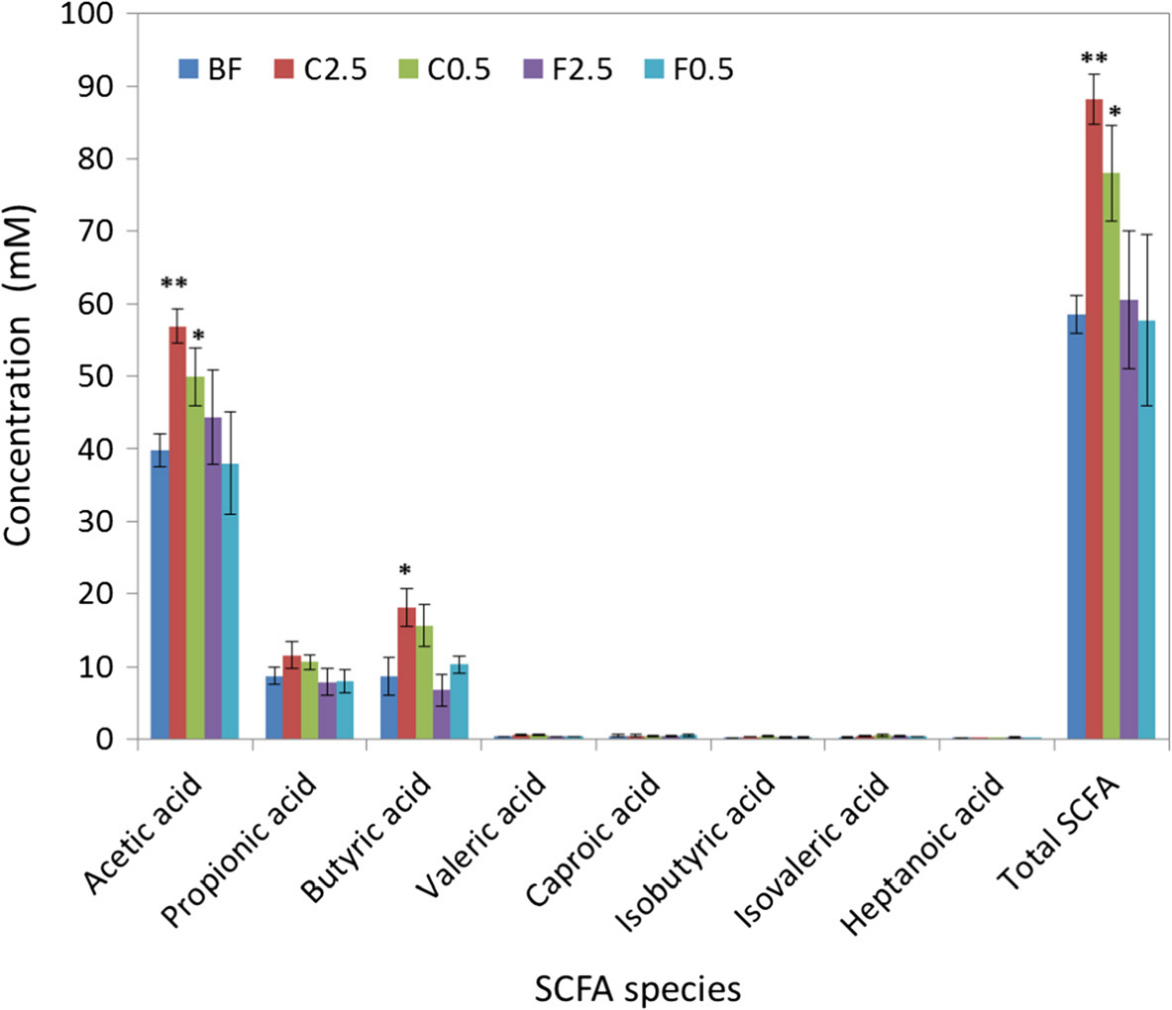
Effect of diets on faecal SCFA concentrations. BF = basal feed; C2.5 = *C. crispus* 2.5 %; C0.5 = *C. crispus* 0.5 %; F2.5 = FOS Inulin 2.5 %; F0.5 = FOS Inulin 0.5 %. SCFA = short chain fatty acid. Data are presented as the mean ± SE. *p < 0.05; **p < 0.01 (versus BF)

Short chain fatty acids (SCFAs) are the main metabolic products of anaerobic bacteria in the GI tract [30]. Therefore, the abundance of intestinal SCFAs indicates the metabolic activity of anaerobic gut bacteria. To investigate the influence of diet on metabolic activity of the gut microbiota, fresh feacal samples from each feeding group were collected, and SCFAs were quantified by GC-FID. Despite their fluctuating abundance among various diet groups, acetic, propionic and butyric acids were found to predominate in all diet groups (Fig. 6). In the C2.5 group, the concentration of these SCFA species increased significantly by 1.4-, 1.3-, and 2.1-fold, respectively as compared to the control group (p = 0.001, 0.04, and 0.01, respectively; Fig. 6). Similarly, acetic and butyric acids significantly increased in the C0.5 group (p = 0.02, 0.03, vs. control, respectively), and the concentration of propionic, n-valeric, iso-butyric, and iso-valeric acids were not significantly affected, as compared to the control (Fig. 6). The total amount of all the tested species of SCFAs was significantly elevated in the C2.5 and C0.5 groups. The concentrations of acetic, butyric, and total acids showed a slight, but not significant, increase in the F2.5 or F0.5 group, as compared to the control (Fig. 6).

### Effects of diets on colonic histo-morphology

**Fig. 7:**
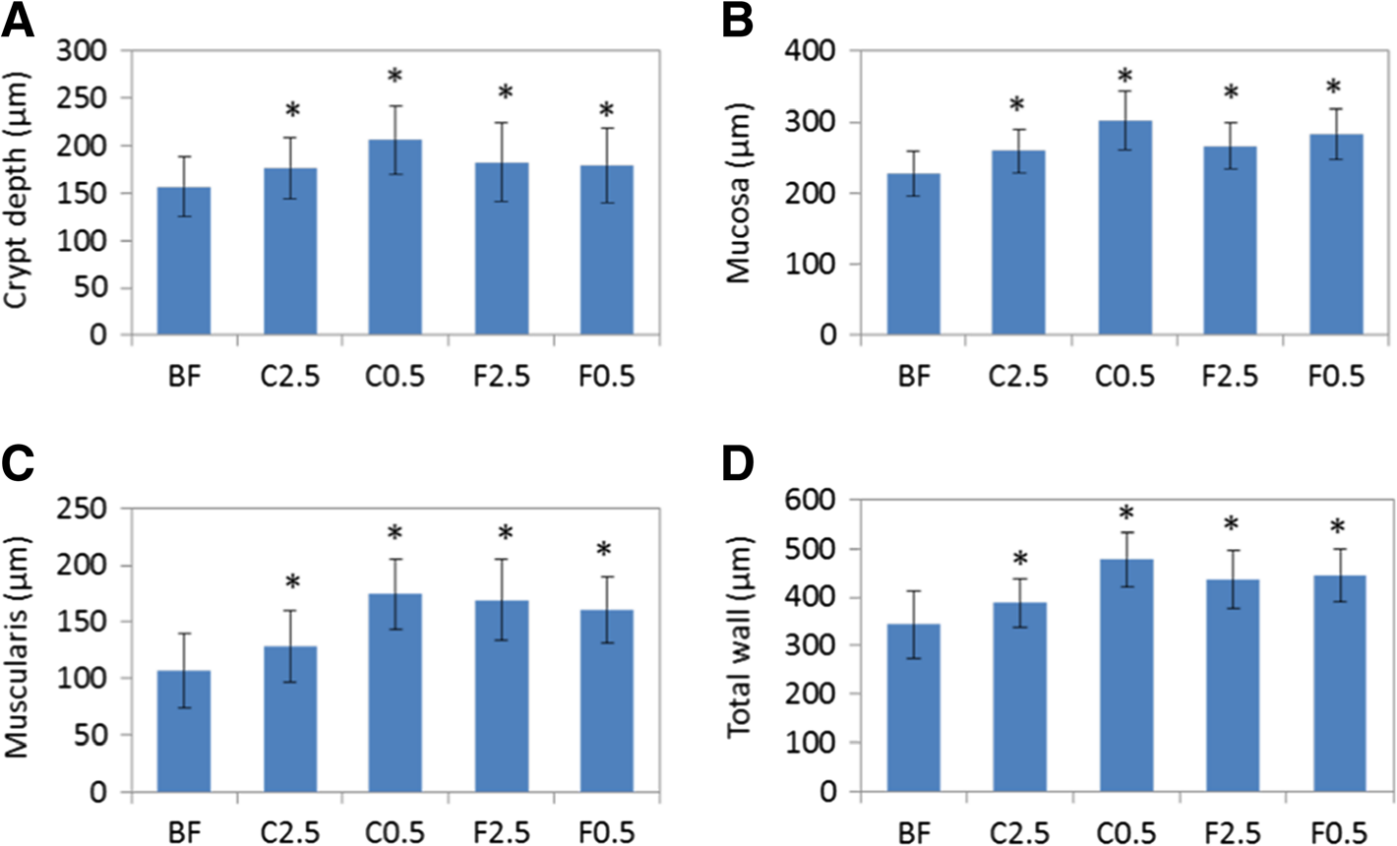
Effect of diets on the histology of rat proximal colon. Shown were changes in the depth of colonic crypt (**a**) mucosa (**b**) externa muscularis (**c**) and total wall (**d**). BF = basal feed; C2.5 = *C. crispus* 2.5 %; C0.5 = *C. crispus* 0.5 %; F2.5 = FOS Inulin 2.5 %; F0.5 = FOS Inulin 0.5 %. Data are presented as the mean ± SD. *p < 0.05; **p < 0.01 (versus BF)

After 3 weeks of dietary supplementation with *C. crispus*, the proximal colon of the young rats showed significant histological changes, as revealed by H&E staining of transverse sections. Dietary supplementation with *C. crispus* at both concentrations resulted in a significant increase in the observed depths of the colonic crypt, mucosa, externa muscularis and colonic total wall (p < 0.05 in all cases), with C0.5 outperforming C2.5 overall (Fig. 7). The C0.5 treatment group, showed increases of 31 %, 33 %, 62.9 % and 38.6 % in these parameters, respectively compared to the control group. The FOS treatment groups (F2.5 and F0.5) also showed significant histo-morphological changes, although the effects were not as great as in the C0.5 group (Fig. 7).

## Discussion

The cultivated red seaweed *C. crispus* contains 50-65 % (on a basis of dry weight) of carrageenan, a sulfated polysaccharide [22]. Like many other dietary fibers, carrageenan is not digested in the upper GI tract and passes through to the large intestine, where it is fermented and utilized by colonic microbiota. In this study, we used a Phylochip assay to monitor colonic microbiota changes resulting from diet supplementation with *C. cripsus*. Phylochip is a comprehensive method to study the microbial communities by using a 16S rRNA gene DNA microarray [31]. Besides being relatively simple and sensitive, Phylochip assay has a major advantage over high-throughput sequencing in that it detects both the presence and the abundance of individual species or other operational taxonomic units (OTUs) [32, 33]. Increases in some beneficial colonic microbiota were observed with *C. crispus* supplementation, such as the well-known probiotic bacterium, *Bifidobacterium breve*. Its increased abundance may have been caused by an enrichment of fermentable substrates as a result of *C. crispus* supplementation. Previous investigations with rats have shown that supplementation of digestion-resistant raffinose in a high-sucrose diet was associated with increased populations of certain species of *Bifidobacterium* [34]. Many other bacterial genera, such as *Legionella*, *Sutterella*, *Blautia*, *Holdemania*, *Shewanella* and *Agarivorans* were also enhanced with *C. cripsus* supplementation. Likewise, microbial decreases were observed in the genus *Streptococcus. C. crispus* fiber (carrageenan) may have been acting as a fermentable substratefor the probiotic bacteria present in the GI tract, thus promoting the growth of the probiotic groups. Further, the probiotics may have outcompeted certain groups of pathogenic bacteria [3]. It is possible that bacterial groups were introduced to the GI tract directly from the surfaces of the seaweed itself (epiphytic/endophytic seaweed bacterial groups), contributing to changes seen in some of the GI tract bacterial communities. However, the microbiota associated with the seaweed remains to be characterized. The relevance of GI tract bacterial community structure on the health of treated rats remains uncertain, because each genus usually consists of multiple species with diverse community functions. The increase in *Bifidobacterium breve* abundance in *C. crispus* fed rats may be associated with beneficial effects, such as immune modulation [9, 16], improvement of consistency and frequency of the stool [4] and prevention of allergic diseases [9, 15]. Other *C. crispus* compounds, including proteins, peptides, lipids, and pigments can also partially contribute to the health of the colon, however these may largely be metabolized in the upper GI tract. Except for *Bifidobacterium* and a few other genera, the abundance of the majority of the 906 OTUs were not markedly affected by either the seaweed or the FOS diet. This is expected since the rats were fed a balanced basal diet and were housed as unstressed test subjects in this current study. Bacterial selectiveness for fermentation substrates [2, 3] can also explain why only a few groups were shifted in relative abundance with *C. crispus* supplementation. Though this experiment was conducted over just 21 days, previous studies have shown 15–21 days of feed intervention to be sufficient to influence colonic microbiota [34, 35]. Besides facilitating favorable changes in gut microbiota, dietary supplementation with cultivated *C. crispus* may also play a role in regulation of pathogenic microbial activity. It has been previously shown that water extracts of this cultivated *C. crispus* also suppressed quorum sensing and the production of virulence factors of the pathogenic bacterium *Pseudomonas aeruginosa* [29]. This selective anti-pathogenic effect may further account for the health benefits in rats with dietary supplementation with cultivated *C. crispus*.

Although the overall composition of the colonic microflora was not altered at the family and phylum levels by *C. crispus* supplementation, a significant increase in the activity of the gut microflora was suggested by the elevated SCFA concentrations recorded in the faecal samples. Possible explanations include: 1) the enhanced metabolism of specific microbial groups, capable of utilizing *C. crispus* fiber as a substrate for probiotic fermentation, resulted in a competitive advantage for these probiotic groups; 2) unmeasured or unclassified microbial groups were able to utilize fermentable *C. crispus* fiber as a substrate for growth.=; 3) the increased abundance of *B. breve* (Fig. 5) may have made a significant contribution to the elevated SCFA concentrations, especially for acetate since this is the major fermentation product of *B. breve* [36]; 4) the fermentation by multiple identified OTUs, whose abundance was enhanced but not to a statistically significant extent, collectively resulted in a pronounced increase in the concentrations of some species and/or the total SCFAs. The major types of gut SCFAs recorded were acetic, propionic and butyric acids [37]. These SCFA’s are reported to have very diverse physiological functions. Butyric acid was shown to be more readily utilized by the colonic epithelial cells than acetic and proprionic acids, glucose and other substrates. Furthermore, butyric acid affects the proliferation and differentiation of colon- and intestine-derived cells; it is consequently important for the health of colonocytes and mucosa [38]. Butyric acid was shown to reduce the incidence and the size of colitis-related tumors in rats [39], which suggests an immune-modulating function of butyrate. All of the three major SCFAs were recently shown to play a role in host-immune regulation. When acetic, propionic or butyric acid was individually, or collectively, supplemented to the diet, germ-free mice showed selective increases in the abundance and functions of the colonic T_reg_ cells, which suppressed inflammation [40]. In the current study, higher concentrations of fecal SCFAs were measured in both the 2.5 % and 0.5 % seaweed supplemented groups, but not in the FOS-supplemented animals. The plasma immunoglobulin IgA and IgG levels were elevated in the C0.5 supplemented group. The C2.5 group shown no such elevation of IgA or IgG. Also colonic histo-morphological parameters were improved to a lesser extent in the C2.5 group than in the C0.5 group (as compared to the control). There is a possibility of decreased absorption of SCFAs by the colonocytes with higher concentrations of dietary *C. crispus* (C2.5 group), due to the high content of water soluble polysaccharide and its gel-forming properties [41], as shown by the increased faecal moisture content recorded in this group.

In addition to colonic epithelial cells, SCFAs are readily absorbed and metabolized in the liver [42]. The uptake of acetic acid by the liver was shown to be enhanced when the supply increased.It has also been reported that butyric acid uptake by the liver was higher than that of propionic acid. Both fatty acids were metabolized and thus barely detectable in the liver under normal physiological conditions [42]. An increased supply of SCFAs in the gut may contribute to an increase in liver weight. This may explain the finding that liver weight was slightly, but not significantly, increased in *C. crispus-*supplemented rats (Fig. 1b). It suggests that a larger amount of SCFAs was produced and utilized by the liver, as compared to the control animals. The overall health status of the animals was not negatively affected by dietary supplementation of 0.5 % or 2.5 % of *C. crispus* or FOS as shown by feed intake (Additional file 2) and the clinical pathology assay of plasma samples prepared from cardiac blood (Additional file 3).

A significant improvement in the colonic morphology of young rats was recorded when fed a diet supplemented with *C. crispus* for 21 days. Andriamihaja et al. [35] found that the morphology of colon epithelial cells was markedly modified in rats after they were fed a high-protein diet for 15 days, which was associated with changes in the abundance of colonic SCFAs, such as propionic and valeric acids, but not acetic and butyric acids. Our observation of elevated faecal moisture content in rats supplemented with 2.5 % *C. crispus* may be related to increased growth of colonic mucosa. Indeed, the dietary bulk content (as reflected by the moisture content of digesta or faeces) tended to positively correlate with colonic mucosal growth (as revealed by colonic mucosal DNA synthesis) [43]. In this study, weaning rats in the rapid growth phase were used. This rapid growth may have further contributed to the dramatic changes in the gut morphology in response to dietary supplementation with *C. crispus*.

## Conclusions

The results of the rat trial presented here suggest a number of beneficial effects from dietary supplementation with cultivated *C. crispus*. Increased abundance of beneficial colonic microbiota with a concomitant decrease in pathogenic microbes was observed. Further, *C. crispus* supplementation resulted in an increase in the concentration of SCFAs, promoted colonic growth and elevated immunoglobulin levels. Thus, cultivated *C. crispus* shows promise as a functional food due to its prebiotic effects.

## Additional files

Additional file 1:
**Effect of diets on the weight of kidney, spleen, and heart.** BF = basal feed; C2.5 = *C. crispus* 2.5 %; C0.5 = *C. crispus* 0.5 %; F2.5 = FOS Inulin 2.5 %; F0.5 = FOS Inulin 0.5 %. Data are presented as the mean ± SD. P > 0.05 (versus BF). (DOC 38 kb) Additional file 2:
**Effect of diets on feed intake.** BF = basal feed; C2.5 = *C. crispus* 2.5 %; C0.5 = *C. crispus* 0.5 %; F2.5 = FOS Inulin 2.5 %; F0.5 = FOS Inulin 0.5 %. Data are presented as the mean ± SD. P > 0.05 (versus BF). (DOC 38 kb) Additional file 3:
**Effect of diets on blood clinical biochemistry in rats.** (DOC 54 kb)

## Abbreviations

FOS: fructo-oligo-saccharide
GI: gastro-intestinal
PCA: principal component analysis
OTU: operational taxonomic unit, which was defined by 16S rRNA gene sequences of > 99 % similarity
HC-AN: hierarchical clustering via average-neighbor method
SCFAs: short chain fatty acids
GC: gas chromatography
FID: flame ionization detector
ELISA: enzyme-linked immunosorbent assay
H&E: hematoxylin and eosin
