# Supplementary material for: Prebiotic effects of diet supplemented with the cultivated red seaweed Chondrus crispus or with fructo-oligo-saccharide on host immunity, colonic microbiota and gut microbial metabolites
Source: BMC Complement Altern Med. 2015 Aug 14;15:279. doi: 10.1186/s12906-015-0802-5 (PMC4535385; doi:10.1186/s12906-015-0802-5)

**Additional file 2.** Effect of diets on feed intake. BF, basal feed as a negative control; C2.5, *C. crispus* 2.5%; C0.5, 0.5%; F2.5, FOS inulin 2.5%; F0.5, 0.5%. F2.5 and F0.5 groups were used as control standards. Data were presented as the mean ± SD. P > 0.05 (versus BF).


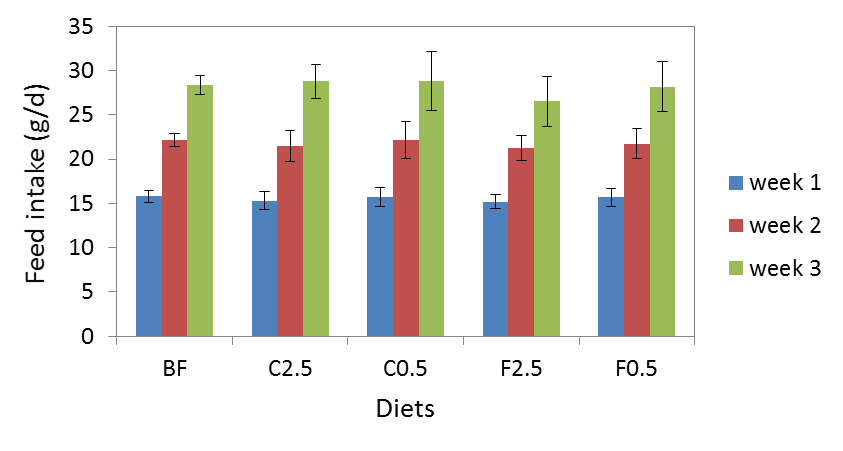

Supplement: Additional file 2: — Effect of diets on feed intake. BF = basal feed; C2.5 = C. crispus 2.5 %; C0.5 = C. crispus 0.5 %; F2.5 = FOS Inulin 2.5 %; F0.5 = FOS Inulin 0.5 %. Data are presented as the mean ± SD. P > 0.05 (versus BF). (DOC 38 kb) [file 12906_2015_802_MOESM2_ESM.doc]
