# Supplementary material for: Prebiotic effects of diet supplemented with the cultivated red seaweed Chondrus crispus or with fructo-oligo-saccharide on host immunity, colonic microbiota and gut microbial metabolites
Source: BMC Complement Altern Med. 2015 Aug 14;15:279. doi: 10.1186/s12906-015-0802-5 (PMC4535385; doi:10.1186/s12906-015-0802-5)
Supplement: Additional file 3: — Effect of diets on blood clinical biochemistry in rats. (DOC 54 kb) [file 12906_2015_802_MOESM3_ESM.doc]

**Additional file 3.** Effect of diets on blood clinical biochemistry in rats.

**Parameter^a^ Feed groups^b^**

BF C2.5 C0.5 F2.5 F0.5

Na^c^

K^c^

Cl^c^

Ca^c^

P^c^

Mg^c^

Urea^c^

Creat.^d^

Gluc.^c^

Chol.^c^

T. bili.^d^

ALP^e^

CK^e^

AST^e^

ALT^e^

T. prot.^f^

Alb.^f^

Glob.^f^

Uric^d^

140.7 ± 2.1

5.6 ± 0.2

96.3 ± 0.6

2.9 ± 0.1

3.6 ± 0.2

1.1 ± 0.1

6.3 ± 0.4

20.0 ± 3.0

14.3 ± 4.3

2.1 ± 0.5

1.3 ± 0.6

380.3 ± 73.1

2449.3 ± 2124.4

185.3 ± 79.4

57.0 ± 13.9

48.7 ± 1.5

29.0 ± 1.0

19.7 ± 1.2

145.0 ± 57.0

142.3 ± 2.5

5.6 ± 1.1

99.7 ± 0.6

2.8 ± 0.1

3.4 ± 0.4

0.9 ± 0.1

5.9 ± 0.6

19.3 ± 1.5

13.9 ± 1.4

2.5 ± 0.1

1.7 ± 0.6

374.7 ± 28.9

1786.3 ± 2544.1

132.0 ± 111.8

52.3 ± 16.4

49.3 ± 2.1

29.3 ± 1.2

20.0 ± 1.0

121.0 ± 78.5

142.0 ± 0.0

6.2 ± 1.1

98.0 ± 2.6

2.9 ± 0.2

3.5 ± 0.4

1.0 ± 0.2

6.5 ± 0.3

21.3 ± 2.5

14.5 ± 4.2

2.5 ± 0.3

1.3 ± 0.6

372.7 ± 43.5

3427.7 ± 3330.7

290.5 ± 48.8

71.0 ± 29.1

52.0 ± 2.7

30.7 ± 0.6

21.3 ± 2.5

164.3 ± 128.4

142.3 ± 0.6

6.7 ± 0.7

98.7 ± 1.2

2.9 ± 0.1

4.0 ± 0.3

1.0 ± 0.0

6.3 ± 0.4

19.7 ± 2.1

12.9 ± 1.2

2.7 ± 0.0

1.3 ± 0.6

334.0 ± 36.3

4324.7 ± 1373.0

259.3 ± 53.3

87.0 ± 28.6

49.3 ± 2.9

29.7 ± 1.5

19.7 ± 1.5

118.7 ± 4.9

142.3 ± 0.6

6.6 ± 0.8

98.3 ± 1.5

3.0 ± 0.1

3.7 ± 0.4

1.0 ± 0.1

5.9 ± 0.3

22.0 ± 1.7

14.9 ± 2.0

2.4 ± 0.5

1.0 ± 0.0

340.0 ± 87.7

1778.3 ± 1578.3

181.7 ± 162.2

68.0 ± 45.1

50.3 ± 2.1

30.3± 1.2

20.0 ± 1.0

106.0 ± 45.3

^a^Na, sodium. K, potassium. Cl, chloride. Ca, calcium. P, inorganic phosphorus. Mg, magnesium. Creat., Creatinine. Gluc., glucose. Chol., cholesterol. T. bili., total bilirubin. ALP, alkline phosphase. CK, creatine kinase. AST, aspartate aminotransferase. ALT, alanine aminotransferase. T. prot., total protein. Alb., albumin. Glob., globulin. Uric, uric acid. ^b^BF, C2.5, C0.5, F2.5, and F0.5, basal diet, 2.5% (dry w/w) *Chondrus crispus*, 0.5% *C. crispus*, 2.5% FOS inulin, and 0.5% FOS inulin, respectively. ^c^, mmol/L. ^d^, umol/L. ^e^, U/L. ^f^, g/L. Data are presented as the mean ± SD. Compared to BF, *P* >0.05 for all parameters. Data for albumin, globulin and total protein were consistent with published reference values for age-matched Sprague-Dawley rats (1).
